# Tissue sampling methods and standards for vertebrate genomics

**DOI:** 10.1186/2047-217X-1-8

**Published:** 2012-07-12

**Authors:** Pamela BY Wong, Edward O Wiley, Warren E Johnson, Oliver A Ryder, Stephen J O’Brien, David Haussler, Klaus-Peter Koepfli, Marlys L Houck, Polina Perelman, Gabriela Mastromonaco, Andrew C Bentley, Byrappa Venkatesh, Ya-ping Zhang, Robert W Murphy, G10KCOS

**Affiliations:** 1 Department of Ecology and Evolutionary Biology, University of Toronto, 25 Willcocks St., Toronto, Ontario, M5S 3B2, Canada; 2 Centre for Biodiversity and Conservation Biology, Royal Ontario Museum, 100 Queen`s Park, Toronto, Ontario, M5S 2C6, Canada; 3 Division of Ichthyology, Biodiversity Institute, University of Kansas, 1345 Jayhawk Boulevard, Lawrence, Kansas, 66045, USA; 4 Laboratory of Genomic Diversity, National Cancer Institute, 31 Center Drive, Frederick, Maryland, 21702-1201, USA; 5 San Diego Zoo Institute for Conservation Research, 15600 San Pasqual Valley Road, Escondido, California, 92027, USA; 6 Theodosius Dobzhansky Center for Genome Bioinformatics, St. Petersburg State University, 8 Viborgskaya Street, St. Petersburg, 194044, Russia; 7 Center for Biomolecular Science and Engineering, University of California Santa Cruz, 1156 High Street, Santa Cruz, California, 95064, USA; 8 Laboratory of Animal Cytogenetics, Institute of Molecular and Cellular Biology, Lavrentiev 10, Novosibirsk, 630090, Russia; 9 Reproductive Physiology, Toronto Zoo, 361A Old Finch Avenue, Scarborough, Ontario, M1B 5K7, Canada; 10 Comparative Genomics Laboratory, Institute of Molecular and Cell Biology, A*STAR, Biopolis, Singapore, 138673, Singapore; 11 Genome 10 K Community of Scientists. http://www.genome10k.org/, Santa Cruz, California, USA; 12 State Key Laboratory of Genetic Resource and Evolution, Kunming Institute of Zoology, Chinese Academy of Sciences, 32 Jiaochang Donglu, Kunming, Yunnan, 650223, China; 13 Laboratory for Conservation and Utilization of Bioresources, Yunnan University, 2 North Cuihu Road, Kunming, Yunnan, 650091, China

**Keywords:** Genome 10K, Sequencing, Vertebrates, Genomics, Tissue sampling, Tissue storage, Cell line, Tissue culture, RNA, DNA

## Abstract

The recent rise in speed and efficiency of new sequencing technologies have facilitated high-throughput sequencing, assembly and analyses of genomes, advancing ongoing efforts to analyze genetic sequences across major vertebrate groups. Standardized procedures in acquiring high quality DNA and RNA and establishing cell lines from target species will facilitate these initiatives. We provide a legal and methodological guide according to four standards of acquiring and storing tissue for the Genome 10K Project and similar initiatives as follows: *four-star* (banked tissue/cell cultures, RNA from multiple types of tissue for transcriptomes, and sufficient flash-frozen tissue for 1 mg of DNA, all from a single individual); *three-star* (RNA as above and frozen tissue for 1 mg of DNA); *two-star* (frozen tissue for at least 700 μg of DNA); and *one-star* (ethanol-preserved tissue for 700 μg of DNA or less of mixed quality). At a minimum, all tissues collected for the Genome 10K and other genomic projects should consider each species’ natural history and follow institutional and legal requirements. Associated documentation should detail as much information as possible about provenance to ensure representative sampling and subsequent sequencing. Hopefully, the procedures outlined here will not only encourage success in the Genome 10K Project but also inspire the adaptation of standards by other genomic projects, including those involving other biota.

## Review

Advances in sequencing technology over the last decade [1-3] have made it feasible to acquire a database for genomes of 10,000 species of vertebrates, analogous to the Human Genome Project. The Genome 10K Project (G10K), which proposes to catalogue whole-genome sequences across living mammals, birds, non-avian reptiles, amphibians, and fishes, will reveal the complex genomic architecture governing the physiology and development of closely and distantly related species [4]. Documenting the dynamic variation of species in a manner permitting detailed comparative genomic and genetic analyses will provide invaluable insight into the fundamental principles driving species’ adaptation to ecological and environmental interactions [5-7]. In this regard, the “Genomics Era” [7] holds promise for new population-genomic approaches to intraspecific biogeography [e.g., [8,9]] and population genetics [e.g., [10,11]] that are imperative to effective biodiversity conservation across species [5,6,12-14]. The process of sequencing, assembling, interpreting, and applying information using whole-genome approaches is starting and quickly building momentum.

As a first step, the G10K Community of Scientists [4] proposes to assemble a collection of tissues and DNA samples representing 10,000 extant vertebrate species. This biospecimen collection will be increasingly valuable the more it is able to standardize procedures for collecting, transporting, and storing high-quality tissue samples. This process, which applies to all genomics projects, is remarkably complex and daunting, especially because many of the existing tissue collections have a history of development, preservation, and storage for different purposes. Many potential resources are not suitable given the requirements of current sequencing and assembly technologies, generally because of insufficient yields of high molecular weight DNA from ethanol-preserved or improperly frozen tissue samples.

Proper collection and preservation of tissues across vertebrate species is fundamental to establishing cell cultures and isolating nuclear and mitochondrial DNA, RNA, and potentially proteomes suitable for genomics, as these materials are susceptible to rapid post-mortem degradation or degradation following tissue-sampling from living specimens. High-quality DNA will facilitate *de novo* assemblies of whole genomes, while viable cell cultures and RNA will be critical for experimental molecular and cell-based investigations, physical mapping of genes onto chromosomes (e.g., fluorescence *in situ* hybridization, radiation hybrid mapping, chromosome flow sorting), transcriptome analyses, and annotation. The standards of material collected for G10K and other projects will vary according to the exigencies of collecting specimens, including ease and method of capture, availability of specimens, feasibility, tissue type, and target quantity and quality. G10K, its contributors, and other researchers will benefit from adopting standardized methods that correspond to their goals of collection. Therefore, we propose standards for sample collection to facilitate and, more importantly, motivate the highest quality, and most broadly useful and valuable samples possible. We also review a range of issues related to selection and documentation of the individual sampled animals including some pertinent legal and ethical considerations.

To help standardize and assess the quality of tissues collected, we propose four categories for classifying the utility of tissues and DNA being prepared and reserved for G10K and similar projects:

· *Four-star* (****): sufficient flash-frozen tissue or immediate extraction of DNA for a minimum of 1 mg of DNA (e.g., enough DNA for at least two whole-genome sequencing attempts) validated by DNA barcoding; multiple tissues suitable for RNA sequencing and transcriptome analysis; and viably frozen tissue pieces and/or cell lines;

· *Three-star* (***): frozen tissue for a minimum of 1 mg of DNA and multiple tissues suitable for RNA sequencing and transcriptome analysis;

· *Two-star* (**): frozen tissue for 700 μg of DNA; and

· *One-star* (*): ethanol-preserved tissue for 700 μg DNA of high or mixed quality (some highly or slightly degraded) and DNA of insufficient quantity (< 700 μg), but of possible value in supplementing whole-genome sequencing efforts of higher quality samples.

These standards have significant implications for the quality and quantity of data for future projects on *de novo* vertebrate genomics. The following text details methods for tissue acquisition and preservation in light of these four standards. At and below the * standard, attempts at producing whole genome sequences are not likely to meet with success without reference genomes and notably greater expense.

### *A priori* considerations

Optimal techniques for acquiring ****, ***, **, and * samples will vary according to species, sex, geographic diversity, and population diversity across the major vertebrate groups [4]. It is critical to consider the individual history of each specimen in order to maximize a reliable yield of tissue, DNA, and RNA. For some species (e.g., mammals and birds), blood may be a source of genetic material, whereas whole specimens may be required to obtain sufficient quantities of DNA in others (e.g., amphibians and non-avian reptiles). At a minimum, the feasibility of each procedure will depend on budget, transport, availability, health of the source-specimen, and the extent of degradation prior to or after sample collection. Appropriately, tissue collection should include back-up procedures (e.g., multiple samples, back-up power supply for freezers, multiple copies of appropriate documentation) whenever possible while minimizing all safety risks, as with any experimental design.

Apart from sample standards, documentation and archiving of permits held by every provider is mandatory for all material collected for G10K. We encourage this for other projects and below consider this universality to be implicit in all references to G10K. Given difficulties in using available museum collections, it will be necessary to acquire fresh material, especially for **** and *** samples. Consequently, all relevant permit and license applications should be prepared and submitted well in advance of tissue collection to allow for review and processing time. Approved written animal care (use) protocols may be necessary from, for example, an Institutional Animal Care Use Committee (IACUC) or Animal Care Committee (ACC), and in some cases animal health permits will be required. All procedures must conform to institutional, local, state, and/or federal guidelines [e.g., [15-19]].

Many countries including the United States are signatories on the “Nagoya Protocol on Access to Genetic Resources and the Fair and Equitable Sharing of Benefits Arising from their Utilization to the Convention on Biological Diversity”, promoting genetic research toward conserving biological diversity [[20], see [21] for text]. Accordingly, equal access and benefit-sharing should be ensured, in addition to complying with regulations protecting general public health, domestic crops, and livestock or control of trafficking of listed species [e.g., [17,22-25]]. In many countries including the United States, Canada, China, Vietnam, and Mexico, research within national parks, nature reserves, marine conservation areas, historic sites or landmarks requires additional research permits (e.g., see [17] for Australia, [26] for USA, [27] for Canada, and [28] for New Zealand). In some cases, research licenses may apply to particular territories (e.g., Nunavut, Canada) or cultural permission may be necessary; for example, consultation with the Maori of New Zealand is requisite for sampling wildlife that may lead to cultural sensitivity [29]. It is necessary to acquire permission for sampling natural populations from the appropriate fish, wildlife, forestry, conservation, and other offices.

Permits and licenses may also be necessary for import and/or export (e.g., [30]; for a list of permits see [31] for Australia, [32] for Canada, [33] for New Zealand, and [34] for USA). These constraints can depend on taxon and country. For example, in Canada, a “Permit to Import Material of Animal or Microbial Origin from Agriculture and Agri-Food Canada” is required for imports and exports of many but not all vertebrate groups [23]. Prior to air travel, check all potential specimens or chemicals for classification as “Restricted Articles” (may not be carried as checked or hand-carried baggage on commercial aircraft) by the International Air Transport Association (IATA) and appropriate “Shippers Declaration for (Non) Dangerous Goods” certificates should be acquired [35]; a list of potentially required documents is available [36]. Compliance with applicable regulations routinely requires declaration of all specimens to customs officials upon arrival after crossing borders. Many large laboratories or institutions and museums already have permits in place and have trained and experienced specialists; researchers are encouraged to seek their help and advice to streamline the permit application process.

### Collecting tissues for G10K

#### Description of specimens for G10K

Careful identification and validation by a specialist is necessary to ensure true subsequent sequencing representation. In cases where cryptic species are likely to exist, sample from the type locality when possible. Species selected for G10K should have established biological relevance for society and the scientific community. Characteristics include extreme phenotypes, phylogenetic uniqueness (hence applications for comparative biology), interests for conservation, and relevance for other scientific studies. Targeted species should allow multiple samples to be collected from one individual (e.g., large body size) for high-coverage sequencing. Smaller amounts of DNA (about 30–100 μg) from several individuals of the same species will support light-coverage sequencing for the discovery of single-nucleotide polymorphisms. Triads (parent–parent–child or sibling–parent samples) will promote further genome exploration and haplotype description.

At least one reference species will also be targeted for 130 vertebrate orders [4] and these should be included among the **** collections. These species will supply high-quality assemblies (thus requiring storage and preservation of more tissue). Reference species will facilitate the assembly of closely related non-reference species. Characteristics of reference species include accessibility to multiple tissues (for transcriptome analysis), samples, individuals and subspecies, as well as the possibility of banking both sexes (assuming chromosomal sex determination). Samples from reference species should also allow for potential chromosomal mapping (karyotyping); the establishment and banking of viable frozen cell cultures is encouraged. Consistent with standards for targeted ordinal representatives, as much information as possible should be collected for all specimens in order to effectively link phenotypes of a particular specimen “type” to its genome (see Lodging of vouchers below).

The sequencing of both sexes (e.g., non-recombining and sex-determining regions) provides important markers and crucial data for evolutionary and biological inferences. In spite of this, few genomic-wide sequences are now assembled for both sexes as current genome sequencing and assembly efforts may be challenging with highly repetitive data characteristic of sex-determining chromosomes (e.g., Y chromosome in mammals or W chromosome in birds). If sex determination is associated with structurally diverged chromosomes, collections from specimens of the heterogametic sex will allow G10K to obtain as much information as possible for each sex; for example, sequencing the heterogametic sex will allow for the development of sex-specific markers (Y or W) for applications in sex-biased dispersal or gene flow relevant to population genetics. Alternatively, sequencing homogametic individuals will be less expensive and double the coverage on the (X or Z) chromosome (e.g., equivalent to that of autosomes) [37]. In this time of short reads and high coverage (> 50 times), the selection of the heterogametic sex seems preferable as is illustrated by the adequate X-chromosome assembly of the male compared to female ferret (D. Jaffe, personal communication).

#### Freshness

All anaesthetization or euthanasia procedures will require *a priori* academic review and should conform to accepted practices as outlined by the IACUC [38], American Veterinary Medical Association Guidelines on Euthanasia [39], or requirements specific to other nations. Handling and use of chloroform, ketamine, pentobarbital, tricaine, clove oil, or taxon-specific methods must follow legal procedures [e.g., [15-19]]. Drugs that potentially alter RNA expression or DNA quality should be avoided [40]. If euthanasia is necessary, collect tissue as soon as possible. When feasible, the whole carcass should be properly stored as a museum voucher specimen, including the skeleton, for future reference.

We recommend collecting tissue opportunistically from live or freshly euthanized specimens for **** and *** standards whenever feasible, while minimizing loss of value of the animal for museum preparations. Wild-caught animals from precise geographical locations, especially type localities of species, are preferred. Noninvasive methods and biopsies will be less damaging to specimens and hence the collected material. Record the general health of all specimens and any obvious parasites. Avoid encysted or parasitized tissues whenever possible. Caution should be exercised when collecting tissue from cancerous or damaged organs to ensure that healthy (versus cancerous) tissue is used as the source for genomic sequencing. Diseased and dying individuals may lead to altered RNA expression, thus affecting transcriptomes.

Tissue from salvaged dead animals will generally not be suitable for either assessment of transcriptomes or the preparation of cell cultures, unless they are exceptionally fresh. These tissues will likely contain only 5–20% of the quality of DNA extractable from fresh tissue [41], resulting in small DNA fragments or high proportions of mitochondrial DNA that are not suitable for the preparation of large-insert libraries, which are preferable for lower-cost sequencing and high-quality genome assembly. Because long-term storage usually leads to a higher likelihood of degradation and contamination, the sampling of sub-surfaces of soft tissues is desirable [42]. The majority of archived museum collections will only allow for ** or * standards due to limited tissue volume and storage in ethanol [43] or other preservatives (see Preserving tissues for G10K). Samples collected from dry mummified specimens are generally not suitable for sequencing and assembly of a *de novo* genome. However, for species where archived tissue or bone is the only available source of DNA (e.g., extinct species), G10K has a special sequencing initiative, where protocols are evaluated on a case-by-case basis (R.E. Green, personal communications). The respective sampling requirements and methods for these specimens are beyond the scope of this paper. In any case, all tissue collected from archived museum collections will require documentation of approvals and links to the archival institutions and specimen data.

#### Selection of tissue for quality and quantity

Sterile methods are critical to the efficacy of G10K material (DNA, RNA, and either cell lines or tissue culture), hence all ****, ***, **, and * collections should be acquired in isolation of other samples using sterile equipment and stored separately to avoid contamination. When collecting a whole specimen, dissect and remove the stomach and intestine to avoid potential contamination of DNA from consumed food items (however, immediately save and/or prepare these tissues for transcriptomes, as discussed below). Never mix or combine specimens; each sample should correspond to a single individual, not a combination of multiple individuals. In particular, sample DNA, RNA, and cell cultures from the same individual whenever possible. Pertinent reagents should be clean and all instruments or containers should be autoclaved prior to use. Ideally, gloves and dissecting equipment should be disposable and changed between collecting samples. Pay special attention to avoid cross-contamination by human tissues.

Quality of collected ****, ***, **, and * standards will vary with quantity of DNA and RNA and the ability to establish viable cell lines, in addition to feasibility. While one sample of DNA from a particular specimen may suffice regardless of tissue, separate samples from separate tissue types are desirable for RNA to achieve high coverage of the diverse transcriptome. Tissue should be sufficient for at least 1 mg of DNA (approximately 1 x 1 x 1 cm^3^) for **** and *** standards. The ** and * standards require about 700 μg of DNA. Although any soft tissue may yield good quality (high molecular weight) genomic DNA, testis provides the highest yield and hence this is the preferred tissue in species having heterogametic or temperature-dependent sex determination. For immature specimens and homogametic individuals (females in the XY system, males in the WZ system), liver is the next best tissue. Because bile salt contamination can affect tissue stability [41], avoid the gall bladder when sampling from the liver and process the sampled tissue as soon as possible. Other soft tissues such as brain, kidney, spleen, heart, and ovary (without eggs) also yield sufficient amounts of DNA but these organs are typically small in size. Liver and other soft tissues (e.g., spleen, pancreas, lung, glands) are generally prone to faster degradation due to higher levels of nucleases, thus harder tissues (e.g., muscle, kidney, heart) may be preferable. Though skeletal muscle provides large amounts of tissue, yields of high molecular weight DNA are small due to the tough nature of muscle fibers.

For large and live specimens, blood is a good source of high molecular weight DNA, and, further, a preferred tissue for constructing large-insert libraries such as bacterial artificial chromosome libraries. Because fishes, birds, non-avian reptiles, and most amphibians have nucleated red blood cells, blood provides a good source of DNA; when red blood cells are non-nucleated, as in mammals and rarely frogs, white blood cells are the source of DNA [41]. About 3 ml of blood from non-mammalian vertebrates can yield up to 1 mg genomic DNA and can be easily collected from medium- to large-size specimens. When possible, separate blood cells from plasma using a centrifuge prior to freezing. Lysing red blood cells in mammals prior to freezing will yield cleaner DNA; ideally, use clean buffy coats without plasma or red blood cells for DNA extractions. Clean (bacterial-free) sperm can also be sampled as additional sources of DNA using “French straws” [41]. Abdominal massages, vibrators, or specialized copulatory devices may allow the collection of ejaculate from at least some non-avian reptiles [44] and birds [45].

Tissues from multiple organs are preferred for RNA sequencing. A range of soft tissues can be targeted for RNA for **** and *** standards (e.g., skeletal muscle, spleen, heart, blood, kidney, stomach, and other parts of the gastrointestinal tract, reproductive organs, liver, brain, eyes, and lung). When applicable, also target venom and scent glands for RNA. On the one hand, abundant contractile proteins, connective tissue, and collagen in skeletal muscle, heart, and skin tissue may result in low RNA yield [2]. On the other hand, bone and brain tissue may be less subject to degradation and thus yield longer fragments of RNA [42]. When possible, transport all tissues collected in the field to the lab in either liquid nitrogen (preferred) or dry ice (where access to liquid nitrogen may be restricted). However, do not subject tissue intended for cell culture to freezing temperatures without using a cryoprotectant (see below) [Additional file 1

#### Tissue cultures and/or cell lines

For tissue cultures (a **** standard), we recommend tissue collected from eyes, though blood and skin can serve as alternatives. For birds, non-avian reptiles, and amphibians, tissue collected from eyes, trachea, gonads, tongue (amphibians) and blood feathers (birds) are robust sources for initiating cell cultures. Viably frozen deep-skin fibroblasts are preferable for most mammals [Additional file 1]; fibroblasts can yield viable cultures without the need for highly specialized culture media or conditions. Specimens which are rich in connective tissues, such as mammalian ear punches or tail snips or avian trachea, yield fibroblast cell lines with a high proliferation level. If there is an organ or tissue of specific interest in a particular species, we recommend collecting viable biopsies of this tissue. Take biopsies using hand-held biopsy punches (2, 4, 6, or 8 mm diameter), forceps, needle and scissors or scalpel blade, or biopsy darts.

Sampling tissue to establish cell cultures may vary slightly from other collection techniques. Sterility is especially important to avoid the introduction of bacteria or fungi, which will inhibit cell growth and prevent establishment of the culture. Sterile tools are essential, even if all that is available is 70% isopropyl (rubbing) alcohol for cold sterilization. For most specimens, it is beneficial to wipe down the biopsy area with alcohol prior to sample collection. In fishes, build-up of mucous on the skin can lead to an increased chance of contaminated cell cultures, necessitating careful wiping of the mucous with sterile gauze prior to sampling [46]. In mammals, hair and fur can be a major source of contamination. Thus, removal of hair by shaving or clipping the area followed by cleaning the skin with gauze soaked in 70% isopropanol prior to sample collection will eliminate or reduce potential contaminants. If shaving is not possible, a thorough rinse with soap and water followed by rinsing with either 70% ethanol or isopropanol for 15 to 20 seconds is sufficient to reduce surface bacteria or fungi. Avoid disinfectants such as chlorhexidine solution because these are too harsh on cells.

Transport biopsies to a laboratory for appropriate processing in a biosafety cabinet. In the field, working in close proximity to a burner will also provide a sterile environment. Ideally, process biopsies for culturing right away without freezing and prepare multiple viable seeding stocks. Additional biopsies should be viably frozen as a back-up where cell lines cannot be established on the first attempt (e.g., due to contamination). If short-term storage or transport is necessary, samples from mammals or other warm-blooded species can be maintained by completely immersing in phosphate buffered saline (PBS) or tissue culture medium, such as Dulbecco’s modified Eagle’s medium (DMEM) or alpha minimum essential medium (MEM). Supplement this with 1% antimicrobial and antifungal antibiotics and hold at room temperature or 4°C. Mammalian skin and ear biopsies stored in tissue culture medium with antibiotics at 4°C have produced viable cultures after 3 weeks (ML Houck, unpublished observation); tissue samples from birds, non-avian reptiles, and amphibians have also produced viable cell lines although storage time-tolerance is less than that for mammals (ML Houck, unpublished observation). Mammalian biopsies can also be stored in CO_2_ independent medium supplemented with 7% fetal bovine serum (FBS) and antibiotics for up to one week. It is possible to transport whole ears (e.g., artiodactyls or carnivores) in cool conditions without immersion into medium for up to one week.

For long-term storage requirements, biopsies should be minced into small pieces (1 mm^2^) using clean cuts of a scalpel blade or fine scissors rather than tearing the tissue apart, transferred to vials containing cryopreservation medium (DMEM or alpha MEM supplemented with 1% antibiotic-antimycotic, 10–20% FBS and 10% dimethyl sulfoxide [DMSO]), and frozen in liquid nitrogen or nitrogen vapour within a dry shipper [e.g., Additional file 2. It is important to agitate gently the vial to assure coating of all pieces with cryopreservative [47]. To ensure viability, biopsies should be subject to gradual freezing (1°C per minute) using commercially available devices (controlled rate freezer, Mr. Frosty®, Stratacooler® etc.) or Styrofoam containers. For field conditions, it is possible to make a vessel that dips into the neck of a liquid nitrogen tank to ensure gradual freezing. If gradual freezing is not feasible, vials containing minced biopsies with cryopreservation medium can be plunged directly into a dry shipper. Lymphocytes from mammalian blood can be isolated and viably frozen using a 10% DMSO solution. Samples from aquatic species are particularly difficult to transport over long distances or times; recent work with biopsy samples from fish detects rapid degeneration in any solution at 4°C and inefficient cryopreservation in freezing medium within a dry shipper (G Mastromonaco, unpublished observation).

Establish cultures following explants or enzymatic digestion of samples [e.g., Additional file 3. Enzymatic digestion typically involves incubation in a collagenase or trypsin solution for 30 minutes to several hours, depending on the tissue type (e.g., incubate cartilage-derived biopsies for one day in a collagenase-hyaluronidase solution [48]). Trypsin can be harsh compared to collagenase thus take care to avoid over-digestion. Explants—where fibroblasts migrate out of tissue pieces that stick to the bottom of a culture flask—may provide a shorter-lived cell line than enzymatic digestion [49], which is particularly relevant to small samples. Culture explants or digested samples using basic cell culture media (e.g., DMEM or alpha MEM for most mammals; 1:1 mixture of alpha MEM and Clonetics^TM^ Fibroblast Growth Medium for carnivores, elephants, perissodactyls [50] and other “difficult-to-grow” mammals; Liebovitz L-15 for fishes [51], and DMEM or Amniomax C-100 for non-avian reptiles [52]) supplemented with antibiotics and serum in incubators with 5–6% CO_2_ at 40°C (birds), 37°C (mammals), 32–33°C (non-avian reptiles), 20–30°C (amphibians), or 15–27°C (fish) [53]. Detailed protocols for cell culturing are available in Additional file 3, [54], and [55].

For the initial phase of primary culture, antibiotics should include an antimycotic such as amphotericin B along with standard penicillin/streptomycin. Gentamycin can also be used to reduce risk of mycoplasma. Once the primary culture is established, sole use of penicillin/streptomycin should be sufficient during passaging. Avoid the long-term use of strong antibiotics. Pay attention to the temperature of culturing, which should be close to the body temperature of the animal (e.g., 37°C for most mammalian cells). For long-term storage, cryopreserve samples at the primary culture stage as well as the early passage stages using freezing medium as discussed above [56]. All G10K cell lines will be stored in culture and stock centers and cell line centers.

#### Lodging of vouchers

All ****, ***, **, and * tissues should be linked to voucher specimens to ensure integrity of tissue specimen identification and future validation of identity. All tissues and vouchers should be lodged and cataloged within a recognized research collection, along with all necessary permits (collection, import, export, Convention on International Trade in Endangered Species of Wild Fauna and Flora, Animal and Plant Health Inspection Service, etc.) and field notes, to ensure positive identification and reproducibility of results. After collecting tissues, vouchers should be prepared either as study skins (most birds and mammals) or fixed in a 3.7x buffered formaldehyde solution and then transferred into 70% ethanol for long-term storage. Large specimens should have formaldehyde injected into body cavities to ensure uniform fixation of muscle, gut cavity and brain tissue. The skull and bones of the animal can be useful for morphological taxonomical purposes. Digital images of vouchers taken while still alive or shortly after euthanasia are important for specimen coloration purposes. If maintaining physical vouchers is not feasible (very large specimens, etc.) photographic voucher images are acceptable as an alternative as long as positive identification is possible. Access to all samples within their storage location should be restricted and guarded to avoid disappearance, accidental thawing, and contamination of specimens [57].

All data associated with the specimen should be collected in the field according to the Darwin Core protocols [58] outlining species name, determiner and determined date, locality information (country, state, county, locality name, and latitude and longitude), sex, age, size, color patterns and morphs, collector(s), tissue type (e.g., muscle, liver, blood, etc.), original preservation (e.g., ethanol, liquid nitrogen, etc.), etc. and reported to the collection in the form of field notes together with any other relevant information (e.g., associated species, habitat, environmental parameters, etc.). Guidelines for recording sample information are available [59].

### Preserving tissues for G10K

All collected ****, ***, **, and * materials (e.g., tissues, nucleic acids, cell lines) should be stored in packages that prevent damage from UV, exposure to light, contamination, and the entry of other chemicals, including liquid nitrogen. Each package should allow enough room for tissue expansion during freezing while minimizing air pockets to prevent drying and degradation. We recommend using sterile Falcon tubes (15 or 20 ml) or plastic cryotubes with secure screw top lids for collection and subsequent storage in liquid nitrogen and freezers. Avoid tubes with pop-off lids; wrap smaller tubes in aluminum foil or place them into larger tubes [60]. Tissue can be stored in plastic bags within ultra-cold freezers for *** and ** standards. Labels should be inside the bag, not written on the outside or on a tag; the latter two can be lost or obliterated, removing the identity of the sample and rendering it useless. Plastic bags should not be stored in liquid nitrogen [60]. In cases of emergency, it is possible to make aluminum foil packages but fold them into air-tight packets in advance and transport flat [60]. Seal the tissues by additional wrapping in heavy-duty aluminum foil prior to storage in liquid nitrogen to avoid package bursting [60].

Preserve all tissues as soon as possible following collection to eliminate water [40] and minimize oxidative degradation or damage of the genomic materials. Whereas more time is necessary to degrade or damage nucleic acids, RNA can degrade rapidly within minutes [2]. For this reason, all preservation methods should consider storage time until the target materials are isolated. Effectiveness of preservation, especially critical for RNA and cell lines, can be enhanced by cutting tissue into small fragments to increase surface area [2,40,42,43,61,62]. However, excess blending or homogenizing of tissue will lead to further degradation, especially of nuclear DNA [41]. We recommend storage of tissue by immediate freezing. Colder is better. The only exception is fresh biopsy specimens from which cell cultures are to be immediately established; for these specimens maintenance at 4°C—not colder—is appropriate. Secondary methods using preservatives such as ethanol or DMSO will yield varying results [43] and it is preferable to avoid them when possible. Techniques that involve minimizing desiccation, FTA® paper, Guthrie cards, vacuum packing, and household methods [40] are unlikely to work for genome-level sequencing.

#### Freezing

Cryopreservation is the most efficient means of preserving genomic material [4,42,57,61,62]. As a **** standard, flash-freezing in liquid nitrogen halts all chemical and biological processes that lead to degradation [40]. This allows for the long-term preservation of viable cells [40,57], and thus DNA, RNA, and proteins [40], provided a cryoprotectant such as DMSO is used. For cell cultures, optimize cryoprotectants according to tissue-type (as above); concentrations should be high enough to protect cells from crystal formation yet dilute enough to avoid chemical injury to cells [41]. Similarly, freezing should allow time for protection from crystal formation while minimizing chemical damage associated with slow freezing [41].

We recommend storing samples below −130°C, the recrystallization point of water [40], or as cold as possible within the laboratory to maximize preservation. Though most expensive to maintain, we recommend flash-freezing tissue for long-term storage as a **** standard. We recommend freezing and storing tissue below −80°C in the laboratory for ***, **, and * standards if liquid nitrogen is not available. Tissue degradation can occur at temperatures between −20 to −80°C [57] and household freezers (−20°C) will be ineffective due to their defrosting (heating) cycles.

#### Non-freezing

Transport and maintenance associated with flash-freezing can be expensive and prohibitively difficult [62,63], potentially limiting **** collections. Ideally, tissues should have all alcohol drained and be transferred to freezers (−80°C) or liquid nitrogen as soon as possible after collection to prevent any further degradation. Alternatively, tissue, blood, and DNA can be stored at room temperature for up to 6 months in DNAgard Tissue® [64], DNAgard Blood® [65], and DNAstable® [66], respectively; these fluid preservatives may be more convenient in field conditions or during transport to a low-temperature freezer. It is possible to place tissue samples for RNA in RNAlater®, another fluid preservative. Clinical studies show no significant difference in RNA yields between room and lower temperatures for up to 3 months, though storage above 25°C can limit RNA yields [62]. RNAlater® is useful for a broad range of tissues and it bears little to no toxicity or flammability [40]. However, it is quite expensive [e.g., [67,68]].

Tissue preserved in ethanol will be considered a * standard, as degradation can still occur during DNA extraction [61]. For these collections, use an optimal ethanol concentration of 95–99% to preserve the collected tissue [40]; it is possible to enhance the effectiveness of 70% ethanol by adding or 1–3% glycerine or 1xTE buffer instead of water [40]. We recommend adding at least three times the ethanol to the volume of tissue [61]; a higher concentration will also be effective [40]. Replace alcohol after 1 to 2 hours to allow diffusion [41] and again after 2 to 3 days to improve preservation, as tissues can retain water during this time [40,61]. It is possible to enhance this method of preservation by transferring the tissue to a lysis buffer for 24 hours prior to DNA extraction [40,61].

#### Shipping

Use couriers for transporting all tissues and specimens between institutions and from the field. In advance of shipping, check courier-specific regulations for transporting animal samples. Equipment for flash-freezing materials may be difficult to access and process [43,62,63] and should be arranged ahead of time. Liquid nitrogen may be acquired from gas and welding suppliers, universities, hospitals, mining operations, military facilities, and other institutions in vacuum-insulated tanks or portable dry-shippers (e.g., used in absorbing spills) [40]. Transport permits are required for liquid nitrogen and dry ice, which classify as “Restricted Articles”. In both cases, confirm updated regulations prior to shipping [69]. Shipping is usually associated with heavy, non-pressurized metal tanks, though it is possible to check these tanks as baggage if they are empty (e.g., during short trips), but at the discretion of the aircraft’s pilot [69]. The packing and insulation of these tanks requires large volumes of space. It is possible to reduce shipping volumes by placing smaller samples in an isolated vacuum-insulated or Styrofoam box of dry ice. Add plastic tubes filled with water should to tanks with few samples [41].

Though ethanol is inexpensive, accessible at field sites, and does not require extensive precautions for field use [62], it is flammable, evaporates quickly, and classifies as a “Restricted Article”. Recent regulations allow for the transport of scientific specimens in small quantities of ethanol (30 ml internal package: 500 ml total) as non-dangerous goods: IATA Special Provision A180 for International Shipping and a letter of interpretation from the Department of Transportation for domestic shipping [35,70,71]. To ship as non-dangerous goods, specimens and tissues are required to be packaged and marked according to these provisions. DNAgard® and DNAstable® reagents are not “Restricted Articles”, thus negating most regulations associated with transport. However, a “Shipper’s Certification of Articles Not Restricted” may still be required [35,36]. We do not recommend using other methods of fluid preservation (e.g., lysis buffers, DMSO) maintained at room temperature for genomic DNA [42,61], though in some cases these methods may be effective when combined with freezing [43].

#### Identification and quality assessment of DNA and RNA

DNA barcoding—where 648 bp on the 5’ end of cytochrome c oxidase subunit I of the mitochondrial region is sequenced [see [69,72,73], and [74] for more information]—can be a standard, inexpensive, and rapid method for confirming species and tissue identity, especially when employing current high-throughput approaches. A standard quality and barcoding [69,72-74] assessment is requisite prior to sending samples for whole-genome sequencing and is especially important for historic samples retrieved from museum collections. Thus, G10K very strongly recommends barcoding as a **** standard. Whenever possible, it is important to consider levels of heterozygosity—estimated during the first round of sequencing—because the appropriate approach to genome sequencing and assembling depends on heterozygosity; sequencing and assembly are easier with lower heterozygosity. Though anticipated sequencing technologies may accommodate DNA of lesser quality and quantity, currently available instruments such as the Illumina HiSeq or Roche 454 require high molecular weight starting material. Constructing libraries using degraded DNA leads to underrepresentation and low rates of insertions. Although the average read lengths of current next-generation sequencers are short (70–400 bp) and next generation sequencers may require a minimum of 200 base pairs for each read [3], it is essential to generate paired-end reads from libraries with different insert sizes, ranging from 100 bp to 150 kb. High molecular weight is particularly required for preparing large insert-size (20 kb to 40 kb) libraries whose paired-end reads provide the critical long-range linkage required for a good genome assembly. It is possible to estimate DNA quality by running 100–200 ng of DNA on a low-density agarose gel with a high molecular-weight marker (with bands over 20 kb). For example, samples can run on a 0.6% agarose gel at 70–90 V for 1 to 3 hours with a λ-Hind III Digest ladder. The major DNA band from the sample should be larger than the 23 kb band in the ladder. For pulsed-field gel electrophoresis, the main DNA band should be 40 kb or more. DNA purity can be subsequently assessed by using standard 260/280, 260/270, and 260/230 ratios. DNA should be free of proteins, polysaccharides, phenol, or other contaminants. It is possible to estimate the actual amounts of double-stranded DNA using a fluorometer (e.g., either Qubit fluorometer or Agilent Bioanalyzer); spectrophotometric evaluations might not provide accurate estimates for double-stranded DNA.

RNA preparations are often contaminated with residual genomic DNA and should be routinely treated with RNAase-free DNAse I followed by phenol-chloroform extraction and ethanol precipitation. The quantity of RNA can be determined by measuring the optical density at 260 nm in a spectrophotometer (an RNA solution with an optical density of 1 at 260 nm contains approximately 40 μg RNA per ml). It is possible to verify the quality of RNA by running approximately 1 μg of total RNA on a 1.2% denaturing agarose gel together with an RNA ladder and ethidium bromide staining. The presence of sharp and bright ribosomal (28 S and 18 S) RNA bands indicates good quality. If RNA has been degraded, ribosomal RNA bands appear fuzzy or smeary; do not use such samples for preparing messenger RNA. Purity (absence of ribosomal RNA contamination), quantity, and size distribution of messenger RNA can be assayed using lab-on-a-chip technology such as the RNA 6000 LabChip® kit with the Agilent 2100 bioanalyzer. This assay is rapid, requires minimal amount of samples (25 to 250 ng/μl), and provides very precise estimates.

Finally, it is necessary to deposit all generated sequences with an open access repository, such as GenBank, and designate the voucher or tissue holding institution along with any publications emanating from the use of tissues to ensure fidelity and linking of all data to the original source organism and allow attribution of the collection. If appropriate, it is important to acknowledge or list as a middle author “Genome 10 K Community of Scientists” (G10KCOS), as we have done herein, so that the community can track the fruits of its work. Sequence and publication information should be included in the relevant database, which ideally should be accessible online.

## Conclusions

**Figure 1:**
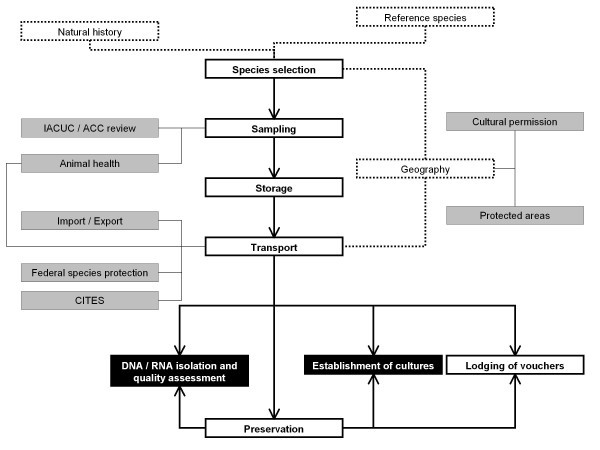
**Considerations (dotted), potential permit requirements (gray) and procedures (bold) in acquiring Genome 10 K materials (highlighted).** Document as much information as possible on permits, sampling events, species identification, and voucher locations.

The G10K project will attempt to follow the standards outlined herein (Table 1; Figure 1): **** (tissue stored in liquid nitrogen for at least 1 mg of DNA, isolated RNA and cell line/tissue cultures), *** (frozen tissue for at least 1 mg of DNA and isolated RNA), ** (frozen tissue for 700 μg DNA), and * (tissue preserved in ethanol for less than 700 μg DNA). We strongly encourage other vertebrate genomic initiatives to adopt this standard. Regardless of standard, it is imperative that all samples collected for G10K follow relevant legal requirements and regulations for their acquisition. The **** standard of tissue collection and preservation is preferred and this will likely require the acquisition of new materials. In contrast, the * standard may not be suitable for genomic-level sequencing given current technological constraints. However, * collections from rare species where sampling may be difficult will still be useful for initial whole-genome sequencing attempts. These guidelines can also be extended to projects focusing on invertebrates (e.g., i5K [75]), plants, and fungi through similar permit, transport, and storage procedures, and particularly considerations where species identification is difficult (e.g., barcoding and archiving procedures). However, some ethical considerations may not be relevant (e.g., animal use protocols in invertebrates are restricted to cephalopods) and specialized protocols for tissue collection (e.g., animals with smaller body sizes) and establishment of viable cultures (e.g., plants) may differ. Accordingly, quantity and quality standards should be established in a similar fashion (e.g. *, **, ***, and ****) at least for invertebrate, plant, and fungi groups. We hope that the methods and procedures discussed herein will not only foster initiatives toward the G10K project, but also contribute to a synchronized understanding of the genetic processes heretofore not available.

**Table 1:** **Tissue standards (*italicized*) for vertebrate genomics corresponding to storage and quality of target materials (bolded)**

	*Four-star*	*Three-star*	*Two-star*	*One-star*
**DNA quantity**				
1 mg	X	X		
> 700 μg			X	
≤ 700 μg ^a^				X
**Target materials**				
Cell lines/tissue culture	X			
RNA	X	X		
DNA	X	X	X	X ^b^
**Specimen type**^c^				
Live/freshly euthanized	X ^d^	X		
Salvaged			X	X
Voucher			X	X
**Storage**				
RNAlater®	X			
DNAgard/DNAstable®	X			
≤ −130°C	X			
≥ −80°C		X		
≥ −20°C			X	
Ethanol				X

^a^Smaller quantities (30 to 100 μg) from multiple individuals of the same species will support light-coverage sequencing for single-nucleotide polymorphism discovery.

^b^High-quality or slightly degraded DNA of small quantities will not likely be sufficient for whole-genome sequencing; these samples may supplement whole-genome sequencing efforts of higher-quality samples.

^c^Standards will vary depending on tissue selection and natural history of the specimen.

^d^Four-star samples should also include reference species for aligning *de novo* sequences of closely related species (see text for more details).

## Abbreviations

G10K, (Genome 10 K); ****, (Four-star); ***, (Three-star); **, (Two-star); *, (One-star); ACC, (Animal Care Committee); IACUC, (Institutional Animal Care Use Committee); IATA, (International Air Transport Association); PBS, (phosphate buffered saline); DMEM, (Dulbecco’s modified Eagle’s medium); MEM, (minimum essential medium); FBS, (fetal bovine serum); DMSO, (dimethyl sulfoxide); G10KCOS, (Genome 10 K Community of Scientists).

## Competing interests

The authors declare that they have no competing interests.

## Authors’ contributions

RWM and EOW conceived the project. PBYW and RWM structured the draft and provided final editing. PBYW coordinated and drafted the manuscript, and synthesized comments provided by all authors. All authors contributed critically important comments. EOW, OAR, WEJ, and RWM contributed to applications in ichthyology, mammalogy, ornithology, and herpetology, respectively. WEJ, SOB, DH, PP, YPZ, and BV contributed to constraints on sequencing genomes. BV contributed to techniques of DNA and RNA extraction and storage. ACB contributed legal and ethical requirements for biospecimen transport. PP and GM provided protocols for tissue acquisition and storage for the establishment of cell lines and tissue cultures; OAR and MH provided protocols in Appendices 1 and 2. All authors read and approved the final manuscript.

## Authors’ information

The G10K project has been coordinated by OAR, SJO, and DH: OAR and SJO are also General Policy Group and Mammal Group members and DH is also a General Policy Group and Analysis Group member. YPZ is a General Policy Group and Mammal Group member. WEJ is Committee Chair for the Mammal Group, and EOW and BV are Committee Co-Chairs for the Fish Group. BV is the Research Director of the Comparative Genomics Laboratory at the Institute of Molecular and Cell Biology, A*STAR, Singapore. RWM is a Committee Co-Chair for both the Amphibian Group and the Non-avian Reptile Group. KPK is a Cancer Research Training Award Fellow at the National Cancer Institute-Frederick and Manager of the G10K database.

## Supplementary Material

Additional file 1 A sample mammalian skin biopsy procedure for subsequent cell culturing. Click here for file

Additional file 2 A sample protocol for freezing tissue biopsy samples prior to subsequent initiation of cell culture. Click here for file

Additional file 3 A sample protocol for the preparation of primary cultures using collagenase digestion. Click here for file
