# Supplementary material for: Tissue sampling methods and standards for vertebrate genomics
Source: Gigascience. 2012 Jul 12;1:8. doi: 10.1186/2047-217X-1-8 (PMC3626508; doi:10.1186/2047-217X-1-8)
Supplement: Additional file 1 — A sample mammalian skin biopsy procedure for subsequent cell culturing. [file 2047-217X-1-8-S1.DOC]

Additional file 1. A sample mammalian skin biopsy procedure for subsequent cell culturing.

Materials

electric clippers (or curved scissors)

straight-edge razors

70% alcohol

sterile gauze

sterile forceps (or needle) and scalpel or biopsy punch

biopsy vial with culture medium and 1% pen/strep fungizone

Protocol

1. Using electric clippers or curved scissors, clip the hair in the area to be sampled, if excessive.
2. Moisten with alcohol.

1. Use a single-edged razor blade and shave the area closely so that all hair is removed.
2. Use a 70% alcohol-drenched sponge to clean the shaved spot **very, very** well.

**Note: Thoroughly cleaning the area with water and soap followed by application of alcohol can also reduce potential contamination.**

1. Have the recipient bottle ready which contains tissue culture medium. Using sterile forceps and a scalpel, grasp a piece of skin from the center of the shaved area and cut off a full thickness of dermis about the size of a small bean or pea.

**Note: Alternatively, one can use a needle to lift the piece of skin and cut underneath with a scalpel or scissors. One can also use a biopsy punch to obtain the biopsy.**

1. Place immediately into recipient bottle and close the cap tightly.

**Note: If the animal is dead, the whole ear, tail, leg, etc. or a large piece of biopsy can be cut and transported to the lab in a plastic bag to be processed in sterile conditions.**

**Note: Discard the scalpel in a safe place. Should you contaminate the knife or scissors accidentally before the biopsy by touching hair, etc., use a new instrument or soak instruments in 70% isopropanol for several minutes before reusing.**
