# Supplementary material for: Tissue sampling methods and standards for vertebrate genomics
Source: Gigascience. 2012 Jul 12;1:8. doi: 10.1186/2047-217X-1-8 (PMC3626508; doi:10.1186/2047-217X-1-8)
Supplement: Additional file 2 — A sample protocol for freezing tissue biopsy samples prior to subsequent initiation of cell culture. [file 2047-217X-1-8-S2.DOC]

Additional file 2. A sample protocol for freezing tissue biopsy samples prior to subsequent initiation of cell culture

Materials

sterile glass plate or petri dish

sterile biopsy vials

medium such as DMEM, alpha MEM or Rosewell Park Memorial Institute Medium

(supplemented with 10% FBS, 1% glutamine, 1% pen-strep fungizone)

DMSO

15- or 50-cc centrifuge tubes

Earle's balanced salt solution (EBSS) supplemented with 1% pen-strep fungizone

sterile 5- or 10-cc pipets

pipettors or pipet bulbs

10-cc syringes

0.2-micron syringe filters

sterile forceps

sterile scalpel

cryo freezing vials (NUNC style, 2-cc capacity)

liquid nitrogen

Protocol (use sterile technique throughout)

1. Take the biopsy sample as described in the Skin Biopsy Procedure above. A 6mm^2^ biopsy will provide enough material for 1 frozen vial.
2. Prepare the freezing medium with 10% DMSO in a centrifuge tube. Prepare enough for 1 cc of freezing medium per vial of tissue to be frozen. Filter the freezing medium through a 0.2-micron syringe filter. Keep the medium on ice or refrigerated.

**Note: Alternatively, use a cryovial with 900μl of medium, add 100μl of 10% DMSO to it, close, and mix well by tapping the end of the tube.**

1. On a sterile glass plate (or Petri dish), mince the tissue biopsy into tiny fragments (1mm^2^) with sterile forceps and a scalpel. Do not let the fragments dry out; keep them moistened with the medium.

**Note: For field preparations where sterile conditions are often not possible, less manipulation with the biopsy will reduce contamination risks. Thus, cleaning the sample site and instruments very well before cutting the biopsy will reduce extra manipulations.**

**Note: If the biopsy sample was not adequately prepared during initial sampling, it may be necessary to rinse the piece in EBSS or medium to remove any excessive amount of hair, fur, etc. This is usually required when working from ear notch samples. Blood vessels and muscle leftovers should also be rinsed off by scraping with closed scissors.**

1. Gather the tissue pieces with forceps and carefully place them into a sterile freezing vial.

**Note: Samples in DMSO should be gradually frozen right away as DMSO is toxic.**

1. Add 1mL of cold freezing medium to each vial. Cap the vial carefully and securely, making sure that the silicone gasket does not bulge out. If capped too tightly, liquid nitrogen will seep inside and cause contamination and/or explosion upon thawing. Gently agitate the cryovial by tapping to expose all tissue pieces to the freeze medium. Ensure the biopsy pieces do not stick to the lid.
2. Label vials with appropriate information (see text), using a water- and alcohol-resistant marker or pencil if a marker is not available.
3. Use a gradual freezing procedure with 1°C per minute cooling rate. Place vials directly into liquid nitrogen for storage.

**Note: Alternatively vials can be placed directly into the liquid nitrogen if gradual freezing is not possible under field conditions.**

**Note: Vials can be stored in liquid nitrogen indefinitely as long as they remain frozen at -130° C or colder the entire time. To establish a cell culture thaw the vial as described below and follow cell culture procedures. Cell culture requires a fully equipped laboratory and cannot be done under field conditions.**

Thawing procedures (use sterile technique throughout)

1. Quick-thaw the sample in a 37° C water bath. Wipe the outside of the vial with alcohol-drenched gauze. Transfer the contents of the vial to a 15cc centrifuge tube containing 10cc of EBSS.
2. Centrifuge at 1000RPM for 10min.
3. Remove as much supernatant as possible. If the pieces look adequately diced, proceed with digestion (begin at step 2 in Additional file 3)**.** If the tissue pieces were frozen in larger fragments than reasonable for adequate digestion (>1mm^2^), remove the washed tissue to a sterile Petri dish and dice the pieces to smaller fragments (step 1 in Additional file 3). Proceed with digestion and cell culture procedures.

**Note: Tissue that has been frozen and thawed in this manner tends to take longer to initiate growth and may in general also produce slow-growing cultures.**
