# Supplementary material for: Tissue sampling methods and standards for vertebrate genomics
Source: Gigascience. 2012 Jul 12;1:8. doi: 10.1186/2047-217X-1-8 (PMC3626508; doi:10.1186/2047-217X-1-8)
Supplement: Additional file 3 — A sample protocol for the preparation of primary cultures using collagenase digestion. [file 2047-217X-1-8-S3.DOC]

Additional file 3. A sample protocol for the preparation of primary cultures using collagenase digestion

Materials

collagenase B 0.5% working solution (Boeringer Mannheim^TM^)

sterile glass plate or Petri dish

sterile scalpel

sterile forceps

sterile 15cc conical tubes

Earle’s balanced salt solution (EBSS)

growth media (see text)

T25 culture flask

T75 culture flask

Procedure (use sterile technique throughout)

1. Using the scalpel and forceps, cut the tissue into small squares (approximately 0.5–1mm^3^). Smaller pieces will digest more rapidly than larger pieces. Use clear cuts. Do not shred or tear the tissue.
2. Gather approximately 10 to 20 pieces together in a clump. Using the forceps, push the pieces to the bottom of a 15-cc conical tube. It is easiest to do this with the pieces in a clump rather than individually.
3. Add approximately 0.3 to 0.5cc of 0.5% collagenase B to the pieces so they are completely covered.
4. Place the tube in a 37°C incubator.
5. About once each hour, gently agitate the tissue pieces in the tube being careful not to let any pieces stick to the side of the tube above the collagenase.

**Note: After 3 to 6 hours, the tissue pieces should appear chewed (crisp edges become “fuzzy”) and the collagenase liquid becomes cloudy with cells and tissue debris.**

1. When the tissue looks sufficiently digested, add 5cc of medium to the tube and pipette the digested tissue pieces and cell pellet in and out of a 5cc pipet to dislodge any cells that are loosely attached to the tissue. Place the entire contents of the tube into a T25 flask.
2. Incubate the T25 flask at 37°C, 6% CO_2_, humidified. This is the primary (1°) flask.

**Note: 37°C is the appropriate temperature for cell culture of most mammalian species. Other species may require different temperatures. See text for details.**

1. Leave the flask undisturbed for 2 to 3 days, then examine the flask for small cell patches under an inverted microscope (there may be only a few patches of 3–10 cells). If a few or no cell patches are seen, replace half the medium and observe again in a few days. If many patches are seen, replace the entire medium.

**Note: Alternatively, 0.5cc of the original (conditioned) medium can be left in the flask as it may enhance cell growth.**

1. When outgrowth is sufficient the cells must be "passed" to a larger flask (secondary culture, 2°). Remove the medium. Rinse with 2cc of EBSS.
2. Remove EBSS. Add 1cc of trypsin and swirl around flask to coat. Place the flask on a warming tray set at 37° C until cells have detached.
3. Add 10cc of medium to the flask and swirl around to immerse all cells into the medium. Transfer cells and medium to a T75 culture flask. This T75 becomes the 2° flask. Re-feed the 1° culture with 5cc of medium. Incubate both 1° and 2° cultures. Cultures should be checked every other day and fed with medium accordingly.

**Note: Cultures may be passed indefinitely and frozen when sufficient numbers of 2° flasks have reached confluency, or harvested for chromosomes when active growth is most evident.**
